# Perceptions of the academic learning environment among occupational therapy students – changes across a three-year undergraduate study program

**DOI:** 10.1186/s12909-022-03291-0

**Published:** 2022-04-25

**Authors:** Linda Stigen, Gry Mørk, Tove Carstensen, Trine A. Magne, Astrid Gramstad, Susanne G. Johnson, Milada C. Småstuen, Tore Bonsaksen

**Affiliations:** 1 Norwegian University of Science and Technology (NTNU), Gjøvik, Norway; 2 Faculty of Health Studies, VID Specialized University, Sandnes, Norway; 3 Norwegian University of Science and Technology (NTNU), Trondheim, Norway; 4 UiT – The Arctic University of Tromsø, Tromsø, Norway; 5 Centre for Care Research North, Tromsø, Norway; 6 Department of Health and Social Functioning, Western Norway University of Applied Sciences, Bergen, Norway; 7 Department of Nursing and Health Promotion, Prosthetics and Orthotics, Faculty of Health Sciences, OsloMet – Oslo Metropolitan University, Oslo, Norway; 8 Department of Occupational Therapy, Prosthetics and Orthotics, Faculty of Health Sciences, OsloMet – Oslo Metropolitan University, Oslo, Norway; 9 Department of Health and Nursing Sciences, Faculty of Social and Health Sciences, Inland Norway University of Applied Sciences, Elverum, Norway

**Keywords:** Higher education, Cross-sectional longitudinal study, Occupational therapy, Course experience questionnaire, Academic learning environment

## Abstract

**Introduction:**

Although the learning environment influences students’ motivation, learning outcomes, and satisfaction with the study program, less is known about how these factors change as the students’ progress through the study program.

**Aim:**

The aim of this study was to examine changes in occupational therapy students’ perceptions of the academic learning environment during their three-year study program and to examine factors associated with the students’ perceptions of the learning environment.

**Materials and methods:**

A longitudinal cohort study was conducted throughout the three-year study program. Data were collected annually using the Course Experience Questionnaire (CEQ). In total, 263 students from six occupational therapy programs participated in at least one data gathering point. The number of participants was 186 in the first year, 168 in the second year and 200 in the third year. Of the 263 students who participated in the study, 87 participated in only one point of data collection, 58 at two points and 118 at all three points of the data collection. Data were analyzed with linear mixed models.

**Results:**

The results showed statistically significant temporal changes on the “Emphasis on independence”, “Good teaching” and “Generic skills” scales. There was a significant decrease in scores from the first to the second year of study and the scores remained at this level in the third study year on both the “Emphasis on independence” and “Good teaching” scales. In addition, associations were found between study effort and educational institution related to the “Appropriate workload” scale, as well as between age and the “Generic skills” scale.

**Conclusion:**

The temporal changes of the students’ perceptions of the “Emphasis on independence” as well as “Good teaching” scales are noteworthy. Both scales indicated a significant decrease in scores, indicating that the students perceived that they were less independent from first to second and third year, as well as a perceived decline in the quality of teaching from first to second and third year. The results of this study are central when planning to facilitate learning, especially related to independence and perceptions of good teaching for students in occupational therapy programs.

## Background

A “learning environment” is a broad term that includes various contextual factors in which students’ learning processes are embedded [1]. More specifically, the academic learning environment refers to the perceived curriculum, instructions, and assessments in higher education programs, such as an occupational therapy study program. Research suggests that the academic learning environment influences students’ motivation [2], learning outcomes [3], levels of self-beliefs and self-regulatory capabilities [4], and satisfaction with the study program [5, 6]. Previous studies suggest that a positive academic learning environment is associated with increased satisfaction, achievement and success in subsequent professional practice [5, 7].

One of the most frequently addressed aspects of the learning environment is what is commonly referred to as “good teaching”. According to literature, good teaching is characterized as informative and interesting lectures [8, 9], high teacher’s expertise and interest in the subject [8, 10–12], as well as with high teacher skills in presentation, preparation and organization [9, 12, 13]. Good teaching has also been described with characteristics such as the perceived quality and effectiveness of instruction [8, 10, 11, 14], fair tests and paper evaluations [8] and the quality of assessment procedures and feedback [11, 12, 15].

However, good teaching is also linked to students’ experiences of well-functioning academic learning environments. Perceptions of different aspects of the academic learning environment, such as heavy workload and inappropriate assessment, have been associated with student’s applying superficial approaches to studying, while perceptions of good teaching influenced them towards deep approaches to learning [16]. Moreover, educators frequently use student-active teaching methods to actively motivate and engage the students [17, 18]. Student-active teaching methods rely on students actively preparing for, participating in, and engaging in lectures and seminars [19]. Research suggests that students develop critical thinking and problem-solving skills through active learning strategies which are prerequisites for clinical reasoning, and decision-making abilities [20, 21]. Critical thinking and problem-solving skills are developed throughout the student’s education and can be labeled generic skills. However, generic skills are comprised of several other skills, such as analytic skills, teamwork, confidence in tackling unfamiliar situations, ability to plan work, and written communication skills [22]. These skills are also recognized as learning outcomes required for working life and studying [23].

A recent study investigating associations between the learning environment and student satisfaction among Norwegian occupational therapy students underline that the learning environment is vital for student satisfaction [6]. The study found that students who scored high on “good teaching”, “emphasis on independence” and “clear goals and standards” also reported overall higher education program satisfaction. The results of that study suggested that strengthening student focused teaching, and the independence of the students, in addition to ensuring that the goals and standards of courses are clear and easy to understand would improve student satisfaction [6]. Student feedback relating to experiences with and assessment of different aspects of the learning environment should inform strategies for changes in the learning environment, to enhance the quality of education programs [1, 6].

The duration of the occupational therapy program in Norway is 3 years and there are six occupational therapy programs in Norway. The distribution of field placements across the three study years differs, but the total time in field placement is similar. The educational institutions are free to adapt the program according to local and regional needs, but all programs build on, and must comply with the national qualification framework [24]. The occupational therapy program in Oslo originated in 1952, Trondheim in 1974, Tromsø in 1990, Bergen in 1993, Sandnes in 2001 and finally Gjøvik in 2013. The programs differ in the number of students, educational platform, admission requirements, and field placement and these differences may contribute to students at different educational programs perceiving the learning environment differently [25]. Thus, investigating Norwegian occupational therapy students’ experiences with the learning environment over time is essential to facilitate a supportive learning environment to ensure the quality of the different study programs.

### Study aim

The aim of this study was to examine changes in occupational therapy students’ perceptions of the academic learning environment during their three-year study program and to examine factors associated with the students’ perceptions of the learning environment.

## Methods

### Design

The study is a longitudinal cohort study and part of a larger inquiry focusing on students’ perceptions of the learning environment (Bonsaksen, Gramstad, Mørk, & Johnson, 2019; Thordardottir et al., 2020; Thygesen et al., 2020), approaches to studying (DaLomba et al., 2020; Gramstad et al., 2020; Mørk et al., 2020; Thørrisen et al., 2020) and academic performance (Bonsaksen et al., 2021) among occupational therapy students in Norway. The data were collected between December 2017 and February 2020 during the students’ first, second and third years of the three-year undergraduate study program.

### Sample recruitment and response rates

**Fig. 1:**
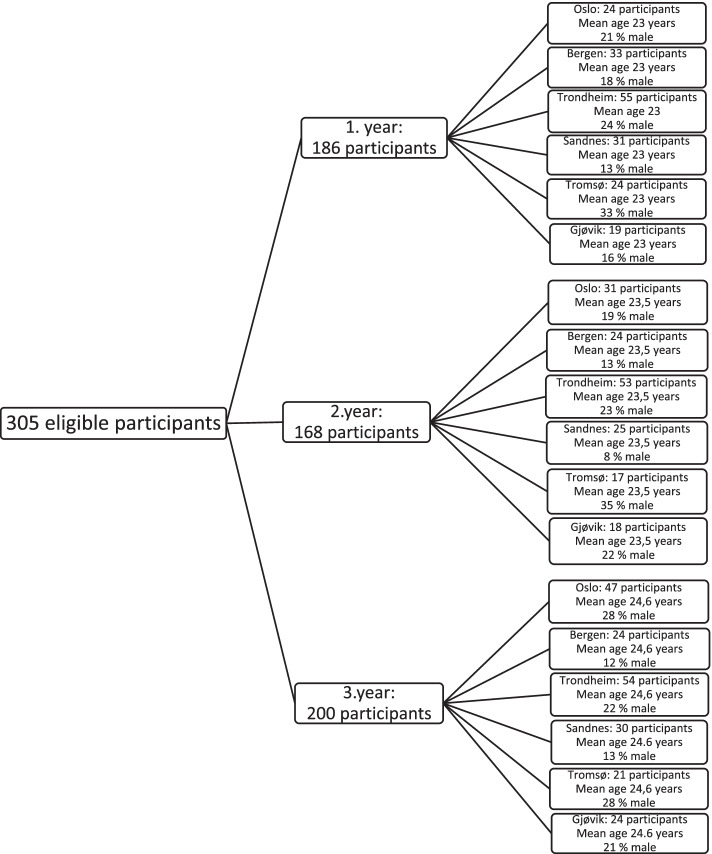
Flow chart of the participants, their university affiliation and demographic characteristics

All students enrolled in the six occupational therapy study programs in Norway were invited to participate at each of the three points of data gathering. One faculty member at each university distributed the questionnaires and consent forms to students. At the commencement of the study 305 students were eligible participants, and 186 first-year students (response rate 61.3%) chose to participate. From the second to the third year of study the response rate increased from 55.1% (168 participants) to 65.6% (200 participants). The students were encouraged by the faculty member, to participate at all three point of data collection and in total, 263 students (response rate 86.2%) participated at a minimum of one assessment. These 263 students were included in the current study sample. Of the 263 students who participated in the study, 87 participated in only one point of data collection, 58 at two points and 118 at all three points of data collection. See Fig. 1 for a flow chart of the participants and the distribution of participants from the six educational institutions.

### Measurement

#### Sociodemographic background and education-related variables

Information regarding sociodemographic background (age and gender), educational institution, and individual study efforts; the latter operationalized as hours spent on independent study during a typical week, was collected as part of the questionnaire.

#### The learning environment

The *Course Experience Questionnaire* (CEQ) (long version) [16, 26, 27], has 37 items distributed onto seven scales: (a) clear goals and standards (5 items), (b) emphasis on student independence (6 items), (c) good teaching (8 items), (d) appropriate workload (5 items), (e) appropriate assessment (6 items), (f) students’ general satisfaction with the course (1 item), (g) generic skills (6 items) and (h) feedback (1 item). The Norwegian translation of the CEQ has been validated [28], and was used in the present study. Table 1 displays example items from each of the employed CEQ scales.

**Table 1:** Scales and example items from the *Course Experience Questionnaire*

Scales	Items
Clear goals and standards	The aims and objectives of this course are not made very clear^a^
Emphasis on independence	Students have a great deal of choice over how they are going to learn in this course
Good teaching	The staff make a real effort to understand difficulties students may be having with their work
Appropriate workload	The sheer volume of work to be got through in this course means you can’t comprehend it all thoroughly^a^
Generic skills	This course has helped develop my ability to work as a team member
Feedback	Feedback on student work is usually only provided in the form of marks and grades
Student satisfaction	Overall, I am satisfied with the quality of this course

The scale ‘Appropriate assessment’ was excluded from the current study

^a^The item has reversed coding

Scores on each item reflect that the participants agree (5), agree somewhat (4), are not sure (3), disagree somewhat (2), and disagree (1). Higher scale scores indicate that the study program is perceived to have (a) clearly established and disseminated goals; (b) high levels of student autonomy and independence; (c) teaching that engages and involves the students; (d) a workload that is not too high; (e) assessment forms that promote and support learning; and (f) support the transfer of content knowledge and skills to the relevant work context. At the first time of measurement, the internal consistency (Cronbach’s α) of each of the scales were (a) 0.73, (b) 0.63, (c) 0.70, (d) 0.69, (e) 0.45, and (f) 0.83. Owing to its low internal consistency, the appropriate assessment scale (e) was not used in the analyses [29]. Instead, we used one item (scored 1–5) assessing the students’ perception of the type of feedback they received from teachers. Higher scores indicated that they usually received varied and extensive feedback, whereas lower scores indicated that they usually received feedback as brief, written comments and/or in the form of grades.

### Statistical analysis

All statistical analyses were performed with IBM SPSS for Windows software, version 26 [30]. The students’ age, sex, and independent study efforts were described using descriptive statistics. Changes over time and at given points for all outcomes were analyzed using linear mixed models (LMM) for repeated measures. LMM allows for estimating trajectories despite missing scores on single occasions, unlike traditional ANOVA for repeated measures analysis. When using LMM, no imputation is necessary as the models use all available data to estimate possible within-subject dependencies. By using all available data, the possibility of selection bias is reduced, as opposed to only completers (students who participated each year) being included. Linear mixed effect models were used to assess the development on each of the five learning environment scales and whether the pattern of development differed by the education institution. Dependencies within individuals were modeled using an unstructured covariance matrix. In addition to time and education institutions, possible confounders (age, gender and study efforts) were entered as fixed effects. The results are expressed as the overall statistical significance of each of the included possible predictive factors (*p*-values) and the estimated means with 95% confidence intervals (CI) at all assessment points. Our results are considered exploratory so no correction for multiple testing was performed and *p*-values < 0.05 were considered statistically significant.

### Research ethics

All methods were carried out in accordance with the institutional guidelines and regulations. The students were informed that completion of the questionnaires was voluntary, their responses would be treated in confidence, and there would be no negative consequences from participating in the study. Their identity was protected by applying codes provided by the staff collecting the data, for the three points of data collection. The code book was kept in locked drawers, separate from the data collected. Approval for collecting, storing, and utilizing the deidentified data was granted by the Data Protection Official at the Norwegian Center for Research Data (NSD) (project no. 55,875). Data were collected directly from the students, based on their written informed consent and in accordance with relevant guidelines and regulations from the granted ethical approval.

## Results

### Participants

The 263 participants were enrolled from the six occupational therapy study programs in Norway, located in Oslo (*n* = 69, 26.2% of the total sample), Trondheim (*n* = 64, 24.3%), Bergen (*n* = 41, 15.6%), Sandnes (*n* = 35, 13.3%), Tromsø (*n* = 28, 10.6%) and Gjøvik (*n* = 26, 9.9%). See Fig. 1 for a flowchart of the participants. The response rates at the educational institutions during the first, second and third time of data gathering are presented in Table 2. A majority was women (*n* = 207 (78.7%) and *n* = 55 (20.9%) were men. One participant did not report the gender. The mean age was 23.0 years during the first data gathering (*SD* = 4.9 years) and the number of hours spent on independent study during a typical week was 8.7 h (*SD* = 6.0 h).

**Table 2:** Learning environment scale scores (estimated marginal means) for each study years

	Overall	1. Study year	2. Study year	3. Study year	Range
Scales	*M* (95%CI)	*M* (95%CI)	*M* (95%CI)	*M* (95%CI)	
Clear goals and standards	17.0 (16.5–17.5)	16.7 (16. 1–17.4)	17.1 (16.5–17.7)	17.2 (16.6–17.7)	5–25 (10)
Emphasis on independence	18.3 (17.7–18.8)	18.8 (18.1–19.5)	18.0 (17.3–18.7)	18.1 (17.3–18.8)	5–30 (12.5)
Good teaching	25.8 (25.0–26.6)	27.1 (26.1–28.1)	24.8 (23.8–25.7)	25.6 (24.6–26.6)	5–40 (17.5)
Appropriate workload	15.4 (14.9–15.9)	15.4 (14.8–16.0)	15.4 (14.8–16.1)	15.4 (14.8–16.0)	5–25 (10)
Generic skills	23.7 (23.2–24.3)	23.0 (22.3–23.6)	23.7 (23.1–24.2)	24.5 (23.8–25.2)	5–30 (12.5)
Feedback	2.2 (2.1–2.4)	2.2 (1.9–2.5)	2.2 (1.9–2.4)	2.2 (1.9–2.6)	5 (2.5)
Student satisfaction	3.7 (3.6–3.8)	3.8 (3.6–4.0)	3.7 (3.5–3.8)	3.7 (3.5–3.8)	5 (2.5)

All the results presented below are estimated using multivariate linear mixed models for repeated measures adjusted for time, education institution and possible confounders (age, gender and study efforts).

### Perceptions of the learning environment

The adjusted students estimated marginal means on each of the learning environment scales, including the one-item scales concerned with “*Feedback”* and “*Student satisfaction”*, are displayed in Tables 2 and 3. Table 3 presents all estimated means according to the students’ educational institutions and are adjusted by the students’ age and study efforts.

**Table 3:** Learning environment scale scores (estimated marginal means) for each of the education institutions

	Oslo	Bergen	Trondheim	Sandnes	Tromsø	Gjøvik
Scales	*M* (95%CI)	*M* (95%CI)	*M* (95%CI)	*M* (95%CI)	*M* (95%CI)	*M* (95%CI)
Clear goals and standards	16.3 (15.5–17.2)	16.5 (15.5–17.6)	17.0 (16.2–17.8)	17.4 (16.3–18.5)	17.9 (16.7–19.1)	16.9 (15.7–18.2)
Emphasis on independence	17.4 (16.4–18.3)	19.1 (17.9–20.3)	18.5 (17.6–19.4)	18.4 (17.2–19.6)	18.2 (16.8–19.5)	18.2 (16.8–19.6)
Good teaching	25.5 (24.1–16.9)	25.1 (23.4–26.8)	26.8 (25.5–28.1)	25.1 (23.4–26.9)	27.8 (25.9–29.7)	24.6 (22.6–26.6)
Appropriate workload	14.4 (13.5–15.3)	15.3 (14.3–16.4)	15.0 (14.2–15.8)	15.1 (14.0–16.2)	16.9 (15.8–18.2)	15.7 (14.5–17.0)
Generic skills	23.4 (22.5–24.3)	24.4 (23.3–25.4)	23.9 (23.1–24.7)	22.6 (21.4–23.7)	24.0 (22.7–25.2)	24.1 (22.8–25.4)
Feedback	2.2 (2.0–2.5)	2.2 (1.9–2.5)	2.2 (1.9–2.4)	2.2 (1.9–2.6)	2.3 (1.9–2.6)	2.3 (2.0–2.7)
Student satisfaction	3.8 (3.5–4.0)	3.5 (3.2–3.7)	3.9 (3.7–4.1)	3.8 (3.5–4.0)	3.8 (3.5–4.1)	3.6 (3.3–4.0)

The estimated marginal means are averaged across the three study years and are adjusted by age and study efforts

Statistically significant changes over the 3 years were found on the “Emphasis on independence”, “Good teaching” and “Generic skills” scales. Regarding other factors associated with students’ perceptions of the learning environment during the study program, the only significant associations were found in relation to the “Appropriate workload” and “Generic skills” scales. See Table 4 for details on the multivariate analysis.

**Table 4:** Overall tests for possible association between selected covariates and learning environment scale scores

Variable	Clear goals and standards	
	*F*	*p*
Age	0.01	0.93
Sex	1.09	0.30
Study efforts	0.30	0.58
Educational institution	1.32	0.26
Time	1.07	0.34
	Emphasis on independence	
Variable	*F*	*p*
Age	3.52	0.06
Sex	0.40	0.53
Study efforts	0.04	0.85
Educational institution	1.19	0.32
Time	3.22	< 0.05
	Good teaching	
Variable	*F*	*p*
Age	0.19	0.67
Sex	0.35	0.55
Study efforts	0.10	0.75
Educational institution	2.06	0.07
Time	11.87	< 0.001
	Appropriate workload	
Variable	*F*	*p*
Age	0.05	0.82
Sex	0.36	0.55
Study efforts	6.75	< 0.05
Educational institution	2.57	< 0.05
Time	0.01	0.99
	Generic skills	
Variable	*F*	*p*
Age	4.52	< 0.05
Sex	0.00	0.96
Study efforts	0.52	0.47
Educational institution	1.48	0.20
Time	12.63	< 0.001
	Feedback	
Variable	*F*	*p*
Age	0.13	0.72
Sex	1.28	0.26
Study efforts	0.46	0.50
Educational institution	0.18	0.97
Time	2.91	0.06
	Student satisfaction	
Variable	*F*	*p*
Age	0.01	0.91
Sex	0.68	0.41
Study efforts	0.01	0.92
Educational institution	1.20	0.31
Time	2.17	0.12

‘Educational institution’ refers to overall differences in scale scores between the education institutions. ‘Time’ refers to linear change in scale scores according to time of measurement

#### Clear goals and standards and feedback

None of the variables were statistically significantly associated with scores on the “Clear goals and standards” nor the “Feedback” scale. There was no change across time, nor were there any overall differences between the other included variables.

#### Emphasis on independence

Related to the scores on the “Emphasis on independence” scale, the multivariate analysis, revealed a statistically significant decrease in scores from the first to the second year of study and the scores remained at this level in the third study year (*p* <  0.05). There were, however, no associations between the other selected variables and the “Emphasis on independence” scale.

#### Good teaching

The multivariate analysis identified a significant drop in scores from the first study year to the second, for the scores on the “Good teaching” scale (*p* <  0.001). Despite an increase in the third year, the scores in the third year were still significantly lower than they were in the first year. There were no other significant associations between the different selected variables and the “Good teaching” scale.

#### Appropriate workload

Concerning the “Appropriate workload” scale, the multivariate analysis showed that the scores remained unchanged from first to the second and third year. However, the scores varied between the education institutions, as the students from Tromsø scored significantly higher than the students from Oslo, Bergen, Trondheim, and Sandnes (*p* <  0.05). The students from Tromsø also scored higher, but not significantly higher, than those from Gjøvik. In addition, those who reported spending more time on the independent study, had lower scores on this scale than their counterparts (*p* <  0.05). No other variables were associated with the “Appropriate workload” scale.

#### Generic skills

The multivariate analysis revealed that the student’s scores on the “Generic skills” scale increased from the first study year to the second and from the second to the third study year (*p* <  0.05). Older students achieved significantly lower scores on “Generic skills”, compared to younger students (*p* <  0.001). None of the other selected included variables were associated with scores on the “Generic skills” scales.

#### Student satisfaction

Related to the “Student satisfaction” scale, the multivariate analysis revealed that the scores slightly decreased over time, although this change did not reach the level of statistical significance. None of the selected included variables were associated with scores on the “Student satisfaction” scale.

See Table 4 for results from the analyses of these possible predictive factors for all analyzed scale scores.

## Discussion

The aim of this study was to examine changes in occupational therapy students’ perceptions of the academic learning environment during their three-year study program and to examine factors associated with the students’ perceptions of the learning environment. Significant changes were found on the “Emphasis on independence”, “Good teaching” and “Generic skills” scales. Regarding other factors associated with students’ perceptions of the learning environment during the study program, the only significant associations were found on the “Appropriate workload” and “Generic skills” scales.

By including three subsequent measurement occasions, we moved the field of study beyond those of previous studies merely comparing cohorts of students and allowed for assessing individual changes in perceptions of the learning environment across time. The scales in which the analysis revealed significant changes will serve as a framework for the following discussion.

### Emphasis on independence

The analysis showed a significant decrease from the first to the second year of study, on the “Emphasis on independence” scale. The scores remained at this level in the third study year. Emphasis on independence is understood as an academic environment that, within the limits of practicality, offers students a degree of choice in developing areas of academic interest, how they learn the material and the forms or modes of assessment [16]. By starting a university education, many students experience challenges with the responsibility of learning being placed on them to a greater extent than in previous schooling [31]. The higher scores in the first year may indicate that the students perceived that they could influence what topics they engage in and focus on more than in the second and third years. There are many obligatory lectures and learning activities in both the first and second study years in the study programs, which the students might perceive as limiting their independence. At the same time, it could be that gradually greater academic maturity and security throughout the course of study gives a greater need for independence in the studies. The scores on the “Emphasis on independence” scale might reflect that the students could not sufficiently meet their need to be independent. Additionally, a potential ‘response shift’ [32] could have occurred. That is; the students scored differently as they adjusted to a new situation, and therefore expected something different from themselves and others.

Evidence-based occupational therapy practice is constantly evolving as new research findings are published [31]. Thus, it is crucial to train students to be independent knowledge seeking occupational therapists during the study program. Therefore, it is important to educate occupational therapy students to have a high degree of independence related to their work. However, the third and final point of data collection took place between December to February in their third year of study. This is before the students embark on their bachelor thesis, which is the largest independent work the students carry out during the 3 years. Thus, it is likely that the results would have been somewhat different had the final data collection taken place after the students had finished their theses. A recent study identified how higher education program satisfaction was significantly associated with higher scores on “Emphasis on independence” scale [6], so it seems important for educational institutions to evaluate how to assure that students can experience a higher degree of independence as they proceed from first to second and third years of studying. Educational institutions aim, after all, to educate responsible and independent occupational therapists who can be innovative and engage in professional development in practice after graduation.

### Good teaching

The changes on the “Good teaching” scale scores revealed a significant drop from the first to the second study year. Despite an increase in the third year, scores were still significantly lower than they were in the first year. The “Good teaching” scale assesses the degree to which students perceive the teaching staff of their courses provide a high level of teaching quality. Specifically, higher scores indicate students perceive adequate feedback on their progress and that the course was presented in an interesting and motivating manner. Additionally, when teaching staff were perceived to make an effort to understand students’ problems and attempt to explain things clearly [16]. One possible explanation for the drop in scores from the first to second year could be that the students might expect more effort from the teaching staff to make sure they understand and comprehend the material, than they receive. Due to students’ expectation of the demands and pressures of working situations, the staff might not provide the guidance and feedback that the students might expect. This clash between expectations and reality might take some time for the students to notice, thus it becomes evident after some time as they progress in their study programs. This could therefore be an explanation for the drop in scores from the first to second year.

Previous research has identified an association between higher education program satisfaction and higher scores on the “Good teaching” scale [6]. Students’ perceptions vary in preferences for a specific learning strategy. However, several studies have highlighted how more active learning methods lead to higher satisfaction and increased examination performance [19]. Additionally, active learning methods contributed to enhance motivation and enjoyment in the learning experience [33]. Therefore, it could be valuable to investigate how more student-active learning can be implemented in the different educational institutions and whether that might lead to a higher score on the ‘good teaching’ scale.

### Appropriate workload

The scores on “Appropriate workload” did not change significantly over time but varied between the educational institutions. The “Appropriate workload” scale assesses the degree to which an academic environment effectively manages students’ workloads and stress by setting a feasible amount of material to be covered, focusing the range of topic areas covered and allowing adequate time frames for work to be completed [16]. Research has demonstrated that when excessive demands are placed on students, they tend to adopt a learning style that involves skimming across the surface of topics without understanding the material. This was recently demonstrated in a study among Norwegian occupational therapy students where lower scores on the “Appropriate workload” scale were associated with higher surface approach scores [34]. Not having an appropriate workload results in poorer academic performance (17) and diminishes the student’s overall enjoyment of the learning process [35]. Higher scores on the “Appropriate workload” scale indicate more that the students perceive acceptable workload levels [16]. The students from Tromsø scored higher on this scale than students from all the other education institutions, perhaps indicating that they viewed their workload as more acceptable compared to the other students. Those who reported spending more time on independent study had lower scores on this scale compared to their counterparts. Therefore, students who spend more time on independent study may have experienced difficulties following the work at campus and thus have to work a lot at home to be able to keep up with the course and the progress of their classmates. On the other hand, the students who follow the work on campus with ease, might be motivated to put more effort into their work at home, even though they might, as a result, score appropriate workload on the lower end of the scale.

### Generic skills

The students’ scores on “Generic skills” increased linearly during all 3 years. The “Generic skills” scale measures the student’s perception related to whether the course contributes to developing generic skills. The types of generic skills assessed with this scale include decision-making skills, problem-solving skills, analytic skills, written communication skills, planning skills, and general ability to address unique problems [22]. There was no variation in scores based on educational institutions, suggesting that the level of these skills developed regardless of educational environment. Whilst discipline-specific skills and knowledge are often crucial to career prospects, there is a consensus that specific knowledge can often become obsolete whereas more generic skills should endure and be applicable in a variety of contexts [22]. The students’ scores increased linearly from first to second and third year, which can be expected as they develop and grow with the tasks and responsibilities they are expected to take on as university students. During both first, second and third year of study, students go to field work in different parts of the health and social system. Those experiences help them develop both generic skills they can utilize in their education, as well as provide them with examples from practice to understand and explain theoretical aspects they encounter during their studies. Older students had lower scores on “Generic skills” compared to younger students likely because older students had some time to develop these skills before starting at the university.

### Feedback and student satisfaction

We did not identify any significant change across time on the “Feedback” and the “Student satisfaction” scales, and none of the variables were significantly associated with these scales. Although the scores on student satisfaction did not differ between institutions or over time, it is worth noting that the scores were reasonably high (3,5–3,9 of 5), indicating that the students were overall quite satisfied with their courses. It is also interesting to note that the student’s scores on the “Feedback” scale were relatively low (2.2 of 5). This indicated that the students perceive that they usually received feedback on their work as brief, written comments and/or in the form of grades. Research has identified barriers to using feedback both from a student and educator perspective. Students might perceive the feedback as not useful, not sufficiently individualized, or too authoritative, and students might not understand the terminology used. It is also important to note students have to be aware of opportunities to receive feedback [36] and lecturers have expressed frustrations when they fail to take note of their feedback [37]. Feedback may be given in situations where students are unaware that feedback is provided, such as in class summaries at the end of terms.

#### Study strength and limitations

A strength of the study is the use of data from all the occupational therapy study programs in Norway, thus making it possible to generalize our results related to both Norwegian and potentially international occupational therapy study programs. However, the sample was modest in size, and the response rates varied between the first, second and third points of data collection, with the highest response rate at the last data collection. Although the response rates differed between study programs, the response rate exceeded 50% of the eligible sample for each data gathering. As comparable general population surveys often obtain much lower response rates (about 30–40%), we consider the response rate in this study satisfactory. Looking at the response rates at the different data collection points it is noteworthy that although Oslo had the highest total number of respondents when adding the three data measurement points, they had a relatively modest number according to their size at the first and second data collection. This indicates that the results in this study might not be representative of the students in Oslo to the degree they are for the students from the other study programs.

Another major strength was the inclusion of three subsequent measurement occasions. Consequently the field of study was extended beyond that of previous studies which had only compared cohorts of students and assessed changes relating to perceptions of the learning environment across time.

This study is based on students’ self-reported data and as self-reported information is a known source of measurement error, this limitation must be considered. Some responses might have been biased by students’ perceptions of a ‘normal’ response. Additionally, a selection bias could have been present initially during participant inclusion. Those who opted to participate may have differed in their attitudes and perceptions (e.g., more motivated and perceiving the learning environment as more positive) than non-participants. However, we do not have any information on the students who chose not to participate in this study due to maintaining students’ privacy. Thus, it is not possible for us to compare our participants to the students that did not participate. Nevertheless, we assume there is no selection bias due to the satisfactory response rate, but we cannot disregard it.

## Conclusion

The aim of this study was to examine temporal changes in occupational therapy students’ perceptions of the academic learning environment during their three-year study program and to examine factors associated with the students’ perceptions of the learning environment. The temporal changes of the students’ perceptions of the “Emphasis on independence” and “Good teaching” scales are noteworthy. Both scales indicated a significant decrease in scores, indicating that the students perceived that they were less independent from first to second and third year, and a perceived decline in the quality of teaching from first to second and third year. As educational institutions aim to educate students to become responsible and independent occupational therapists by the time they graduate, it is important to be aware of these results and facilitate students working more independently as they proceed throughout their education. It is also important to review the quality of the learning activities, especially from 1st to 2nd year, as the students indicated a decrease on the “Good teaching” scale.

The results of this study should be used to facilitate learning among occupational therapy students, especially related to independence and perceptions of good teaching. However, it could be of value to further investigate perceptions of the learning environment with a qualitative approach to get a more in-depth understanding of the student’s experiences of the learning environment and its influence on their learning processes.

## Data Availability

The datasets generated and/or analysed during the current study are not publicly available due to the ongoing analysis and research process but are available from the corresponding author on reasonable request.
